# Histone deacetylase 1 plays a predominant pro-oncogenic role in Eμ-*myc* driven B cell lymphoma

**DOI:** 10.1038/srep37772

**Published:** 2016-11-25

**Authors:** Vincent Pillonel, Nina Reichert, Chun Cao, Marinus R. Heideman, Teppei Yamaguchi, Gabriele Matthias, Alexandar Tzankov, Patrick Matthias

**Affiliations:** 1 Friedrich Miescher Institute for Biomedical Research, Novartis Research Foundation, 4058 Basel, Switzerland; 2 Faculty of Sciences, University of Basel, 4031 Basel, Switzerland; 3 Pathology Institute, University Hospital Basel, 4031 Basel, Switzerland

## Abstract

The two histone deacetylases (Hdacs), Hdac1 and Hdac2, are erasers of acetylation marks on histone tails, and are important regulators of gene expression that were shown to play important roles in hematological malignancies. However, several recent studies reported opposing tumor-suppressive or tumor-promoting roles for Hdac1 and Hdac2. Here, we investigated the functional role of Hdac1 and Hdac2 using the Eμ-*myc* mouse model of B cell lymphoma. We demonstrate that Hdac1 and Hdac2 have a pro-oncogenic role in both Eμ-*myc* tumorigenesis and tumor maintenance. Hdac1 and Hdac2 promote tumorigenesis in a gene dose-dependent manner, with a predominant function of Hdac1. Our data show that Hdac1 and Hdac2 impact on Eμ-*myc* B cell proliferation and apoptosis and suggest that a critical level of Hdac activity may be required for Eμ-*myc* tumorigenesis and proper B cell development. This provides the rationale for utilization of selective Hdac1 and Hdac2 inhibitors in the treatment of hematological malignancies.

Histone deacetylases (Hdacs) belong to a family of 18 enzymes that remove acetylation marks on lysine residues of histone and non-histone proteins^1^. Hdacs modify the epigenome through deacetylation of histone proteins, thereby inducing chromatin condensation leading to transcriptional repression^2^,^3^. They also act on an increasing number of non-histone substrates, nuclear or cytoplasmic, and therefore impact on multiple cellular functions^4^,^5^. Human Hdacs (HDACs) have been reported to have altered function and expression (usually overexpressed) in a wide range of human cancers^6^,^7^,^8^,^9^ and have been considered attractive pharmacological targets for cancer therapy. HDAC inhibitors (HDACis) have potent antitumor activity in hematological and solid malignancies, mainly by inducing apoptosis, inhibiting cell cycle progression and cellular differentiation^10^,^11^. Currently, four pan-HDACis, (targeting class I and/or class II HDACs^12^) are approved for the treatment of T cell lymphoma and multiple myeloma^13^,^14^,^15^,^16^ and several others are in clinical trials for various cancers, including B cell malignancies (reviewed by^9^). However, it is unclear which HDAC isoforms are crucial for tumor cell growth and/or survival, and whether selective HDAC inhibition might have comparable therapeutic benefit with less toxicity compared with broad-spectrum HDACis^2^,^17^.

Although the two class I Hdacs, Hdac1 and Hdac2, have been shown to be implicated in proliferation of cancer cells and to play an important role in hematological malignancies^9^,^18^,^19^,^20^,^21^,^22^,^23^, their exact functions in the different cancer types remains elusive. Hdac1 has been shown to have opposing tumor-suppressive as well as tumor-promoting functions in tumorigenesis and in tumor maintenance, respectively^24^. Numerous studies in different cell types, including B cells, demonstrated that these two enzymes have largely redundant functions during normal development and malignant transformation^25^,^26^,^27^,^28^,^29^,^30^,^31^,^32^. Some studies reported a dose-dependent function of Hdac1 and Hdac2 in some cell types, including T cells and epidermal cells^33^,^34^.

In view of these observations, we assessed the functional role of Hdac1 and Hdac2 in the development and progression of Eμ-*myc* driven B cell lymphomas. Eμ-*myc* transgenic (tg) mice overexpress the *c-myc* oncogene in B lymphocytes and develop multicentric lymphomas associated with leukemia^35^,^36^,^37^. We investigated the impact of B lymphocyte-specific deletions of combination of *Hdac1* and *Hdac2* alleles using targeted conditional deletion with the *mb1-cre* recombinase^30^ in Eμ*-myc* mice. Here, we show that Hdac1 and Hdac2 have tumor-promoting roles in both Eμ*-myc* tumorigenesis and tumor maintenance. This study reveals that *Hdac1* and *Hdac2* have a gene dose-dependent pro-oncogenic role in Eμ-*myc* tumorigenesis, with a predominant role of *Hdac1*.

## Results

### Hdac1 and 2 have no tumor suppressor functions in B cells

**Figure 1:**
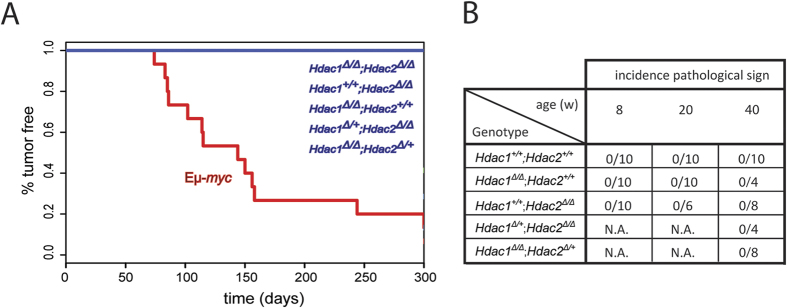
Hdac1 and Hdac2 have no tumor suppressor function in B cells. **(A)** KPLM tumor-free survival curves for 15 age-matched mice are shown with indicated genotypes. Eμ-*myc* tg mice are shown as control. Mice were monitored over a period of 300 days for tumor onset and sacrificed when they reached termination criteria (see Material and Methods). **(B)** Table summarizing histopathological analysis from spleen and lymph nodes of Hdac1 and/or Hdac2 KO mice with indicated genotypes at 8, 20, and 40 weeks. n **= **4–10 as indicated, N.A. for not analyzed.

Previous studies reported that T cell-32,33 and epidermal cell-34 specific ablation of *Hdac1* and *Hdac2* alleles unexpectedly leads to spontaneous tumor formation. Therefore, we first investigated whether ablation of Hdac1 and Hdac2 in B cells also induces tumor development. For this we generated B cell-specific deletions of different combinations of *Hdac1* and *Hdac2* alleles *in vivo* (Supplementary Figure 1A) and monitored mice for tumor development over a period of 300 days by the Kaplan-Meyer (KPLM) method. Interestingly, in contrast to previous observations in T cells, ablation of *Hdac1* and/or *Hdac2* in B cells did not lead to spontaneous tumor development (Fig. 1A). Eμ-*myc* tg mice were used as controls and developed tumors as expected (Fig. 1A; Supplementary Figure 2D). We then performed histopathological analysis from the mice lacking *Hdac1* and/or *Hdac2* to verify the absence of malignant phenotypes. Consistent with the absence of visible and palpable tumors in the KPLM analysis, we did not detect any pathological signs in *Hdac1* and/or *Hdac2* KO mice at 8, 20, and even 40 weeks in the spleen, lymph nodes, or thymus (Fig. 1B). Taken together, our results indicate that Hdac1 and Hdac2 do not have a tumor suppressor function in B cells.

### Eμ-*myc* tumorigenesis is *Hdac1* and *Hdac2* gene dose-dependent

**Figure 2:**
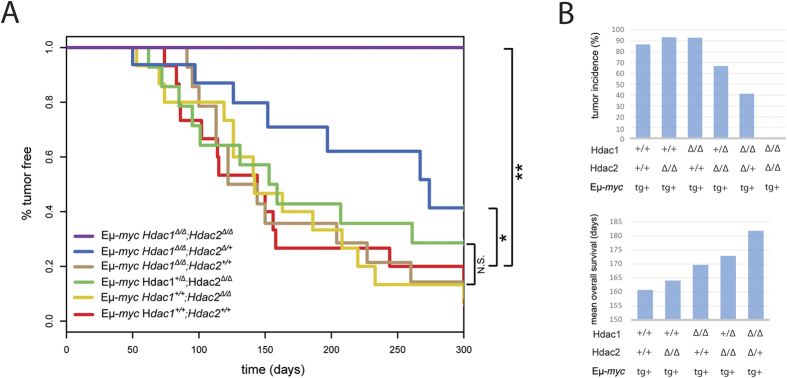
Eμ-*myc* tumorigenesis is *Hdac1* and *Hdac2* gene dose-dependent. **(A)** Kaplan-Meier tumor-free survival curves are shown for 15 age-matched mice with indicated genotypes. The log-rank test was used to determine the level of significance between curves in the KPLM plots. Significant differences between genotypes are indicated, *p < 0.05; **p < 0.01. N.S., not statistically significant. **(B)** Tumor incidence (%; upper panel), and mean overall survival (days; lower panel), according to indicated *Hdac1* and *Hdac2* genotypes. Bar plots show values extracted from panel A.

We next investigated the effect of *Hdac1* and *Hdac2* ablation in the Eμ-*myc* cancer background, and in particular whether they have tumor suppressive or tumor promoting functions during Eμ-*myc* tumorigenesis. We previously reported that concomitant ablation of *Hdac1* and *Hdac2* in non-transformed B cells induced a cell cycle block and apoptosis^30^. We therefore hypothesized that similar ablation of *Hdac1* and *Hdac2* might also have this effect in malignant Eμ-*myc* tg B cells which overexpress the strong *c-myc* oncogene. We crossed mice with B cell-specific deletions of different combinations of *Hdac1* and *Hdac2* alleles with Eμ-*myc* mice in order to obtain mice having the Eμ-*myc* tg in different backgrounds with respect to *Hdac1* and *Hdac2* (Supplementary Figure 2A). Eμ-*myc* mice overexpressed the *c-myc* oncogene in all B lymphocytes, as expected (Supplementary Figure 2B,C), and developed multicentric lymphomas (Supplementary Figure 2D), as previously reported^35^,^36^,^37^. We then monitored tumor-free survival by KPLM analysis in mice having different combinations of *Hdac1* and *Hdac2* alleles. Interestingly, we observed that mono-allelic expression of *Hdac2* in *Hdac1-*deficient Eμ-*myc* B cells (*Hdac1*^***Δ/Δ***^*; Hdac2*^***Δ/*****+**^) resulted in delayed tumor development, whereas complete *Hdac1* and *Hdac2* deletion (*Hdac1*^***Δ/Δ***^*; Hdac2*^***Δ/Δ***^) prevented tumorigenesis altogether (Fig. 2A). We observed a gradual decrease in tumor incidence and concomitant gradual increase in mean overall survival upon deletion of combinations of *Hdac1* and *Hdac2* alleles (Fig. 2B). Importantly, these findings demonstrate that Hdac1 and Hdac2 have pro-oncogenic roles in tumorigenesis of Eμ-*myc* mice, in contrast to other cancer models such as acute promyelocytic leukemia (APL)^24^. These findings further indicate that Eμ-*myc* tumorigenesis is *Hdac1* and *Hdac2* gene dose-dependent. We observed that a critical level of Hdac1 and Hdac2 is required for tumorigenesis. Moreover, Hdac1 and Hdac2 are not completely redundant; we observed that Hdac1 functions as the dominant protein (Fig. 2A,B).

### Complete *Hdac1* and *Hdac2* ablation prevents Eμ-*myc* tumorigenesis

**Figure 3:**
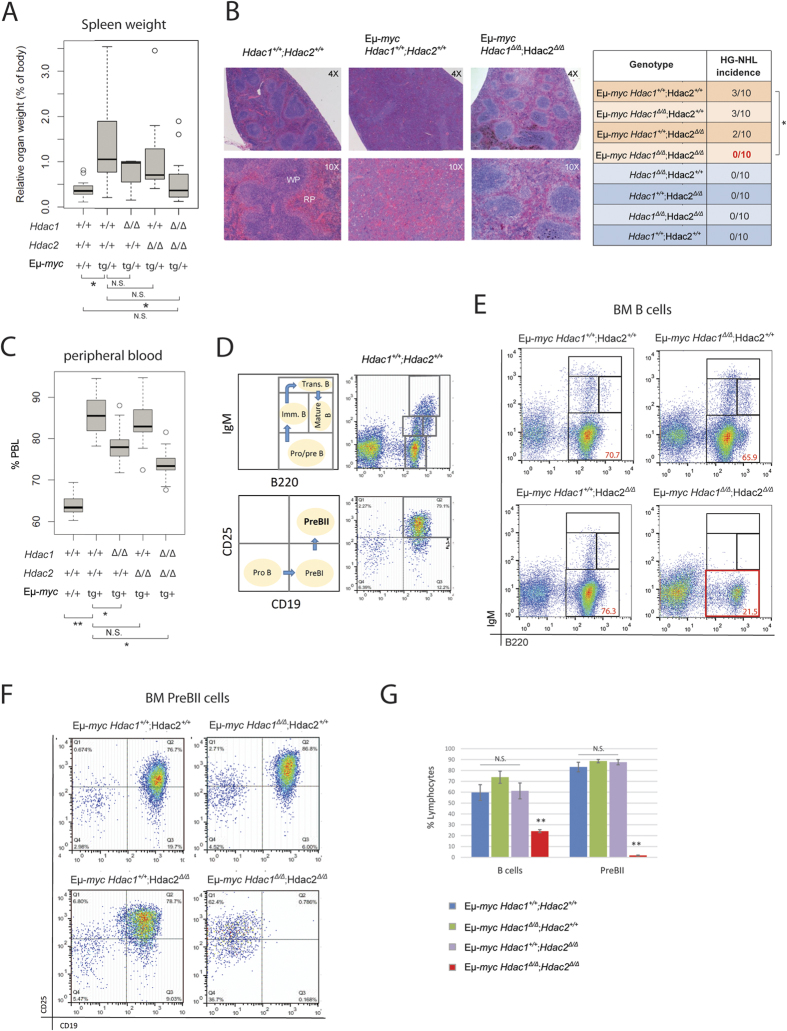
Complete Hdac1 and Hdac2 ablation prevents Eμ-*myc* tumorigenesis. All experiments were performed in 8-week-old mice. **(A)** Boxplot shows relative spleen weight (% of body weight) of mice with indicated genotypes. *p*-values were generated using Wilcoxon Signed-Rank Test (n **= **11). **(B)** Representative pictures from histopathological analysis of hematoxylin and eosin stained spleen sections of Eμ-*myc* mice with indicated genotypes and healthy *Hdac1*^**+/+**^*; Hdac2*^**+/+**^ control. Original magnification of 4X and 10X as indicated (left panel). Pathological findings of HG-NHL were scored in spleen and lymph nodes and summarized (table, right panel, n **= **10). *p*-value was calculated using Student unpaired 2-tailed *t* test. **(C)** Blood analysis with automated blood analyzer. Percentage (%) of PBL of indicated genotypes (n ≥ 10). *p*-value calculated with Wilcoxon Signed-Rank Test. **(D–F)** Eμ-*myc* BM cells from mice with indicated genotypes were stained with B cell surface marker-specific antibodies, including B220, IgM, CD19 and CD25, and analyzed by flow cytometry. **(D)** Schematic representation of wild-type BM profile: B220/IgM to distinguish between Pro/preB (B220^+^; IgM^−^), immatureB (B220^low^; IgM^+^), transitional B (B220^+^; IgM^+^) and mature B (B220^high^; IgM^+^) cells (upper panels). CD19/CD25 to identify PreBII cell subset (B220^+^;CD19^+^; CD25^+^; lover panels). **(E)** Representative flow cytometry dot plots of B220/IgM staining gated on total BM lymphocytes. Gated regions indicate B cell subsets of interest with frequency in percent. **(F)** Representative flow cytometry dot plots showing PreBII lymphocytes subsets. **(G)** Quantification of flow cytometry analysis from (**E,F**; n **= **4–6 biological replicates). Average percentage of B cells (B220^+^) and PreBII cells represented with s.e.m. Statistical analysis was performed with Student unpaired 2-tailed *t* test. Significant differences in means between genotypes are indicated, *p < 0.05; **p < 0.01. N.S., not statistically significant.

The foregoing findings demonstrate that complete *Hdac1* and *Hdac2* deletion (*Hdac1*^***Δ/Δ***^*; Hdac2*^***Δ/Δ***^) prevents tumorigenesis (Fig. 2). To determine how the lack of Hdac1 and Hdac2 in B cells impacts Eμ-*myc* tumorigenesis, we analysed 8-week old mice by first measuring spleen weight, since Eμ-*myc* mice typically have splenomegaly. We found that only complete ablation of both enzymes (*Hdac1*^***Δ/Δ***^*; Hdac2*^***Δ/Δ***^), but not ablation of either Hdac1 (*Hdac1*^***Δ/Δ***^*; Hdac2*^**+/+**^) or Hdac2 (*Hdac1*^**+/+**^*; Hdac2*^***Δ/Δ***^) alone, prevented spleen enlargement (Fig. 3A). We next performed histopathological analysis from spleen (Fig. 3B). As expected, some Eμ-*myc* mice displayed high grade non-Hodgkin’s lymphomas (HG-NHL, roughly corresponding to Burkitt’s lymphoma in humans), as described previously^35^,^38^. Importantly, no *Hdac1*^***Δ/Δ***^*; Hdac2*^***Δ/Δ***^ Eμ-*myc* mice had HG-NHL in spleen and lymph nodes, whereas ablation of either *Hdac1* or *Hdac2* alone did not prevent HG-NHL development (Fig. 3B, table). We next measured circulating peripheral blood lymphocytes (PBL) using an automated blood cell analyzer. Eμ-*myc* mice had significantly elevated PBL compared to control wild-type mice (Fig. 3C). Interestingly, we observed that *Hdac1*^***Δ/Δ***^*; Hdac2*^***Δ/Δ***^ Eμ-*myc* mice had significantly lower PBL counts, compared to control *Hdac1*^**+/+**^*; Hdac2*^**+/+**^ Eμ-*myc* mice, and hence reduced leukemia burdens (Fig. 3C). Ablation of *Hdac1* (*Hdac1*^*Δ/Δ*^*; Hdac2*^**+/+**^), but not *Hdac2* (*Hdac1*^**+/+**^*; Hdac2*^*Δ/Δ*^) in Eμ-*myc* mice, significantly reduced PBL counts at 8 weeks (Fig. 3C). However, this phenotype is transient, since we did not see any effect in PBL counts in older (10 and 20 week old) *Hdac1*^*Δ/Δ*^*; Hdac2*^**+/+**^ Eμ-*myc* mice (Supplementary Figure 4). Hence, we conclude that ablation of *Hdac1* (*Hdac1*^*Δ/Δ*^*; Hdac2*^**+/+**^) alone has no major impact on Eμ-*myc* tumorigenesis. Finally, we performed an analysis of the bone marrow (BM) of Eμ-*myc* mice by flow cytometry. We performed immunofluorescence stainings with B cell-surface-marker-specific antibodies including B220, IgM, CD19 and CD25 to identify the different B cell populations in the BM (Fig. 3D). Eμ-*myc* mice displayed blasts at the Pro/PreB cell stage that dominates the BM, as previously reported^39^. Consistent with the data outlined above, ablation of *Hdac1* (*Hdac1*^***Δ/Δ***^*; Hdac2*^**+/+**^) or *Hdac2* (*Hdac1*^**+/+**^*; Hdac2*^***Δ/Δ***^) alone had no effect (Fig. 3E,F). However, ablation of both *Hdac1* and *Hdac2* (*Hdac1*^***Δ/Δ***^*; Hdac2*^***Δ/Δ***^) prevented the appearance of such Eμ-*myc-*induced B cell blasts at the Pro/PreB stage (B220^+^; IgM^−^; Fig. 3E), more specifically at the PreBII cell stage (B220^+^;CD19^+^;CD25^+^; Fig. 3F). Altogether, these results indicate that ablation of *Hdac1* and *Hdac2* (*Hdac1*^***Δ/Δ***^*; Hdac2*^***Δ/Δ***^) prevents Eμ-*myc* tumorigenesis. However, quantification of flow cytometric analysis revealed that *Hdac1*^***Δ/Δ***^*; Hdac2*^***Δ/Δ***^ Eμ-*myc* mice had significantly reduced B cell numbers and almost no PreBII cells (Fig. 3G). These findings suggest that the effect of complete *Hdac1* and *Hdac2* ablation on tumorigenesis could be due to a B cell developmental defect at the PreBII cell stage.

### Conditional ablation of *Hdac1* and *Hdac2* in Eμ-*myc* tumor cells delays tumor appearance *in vivo*

**Figure 4:**
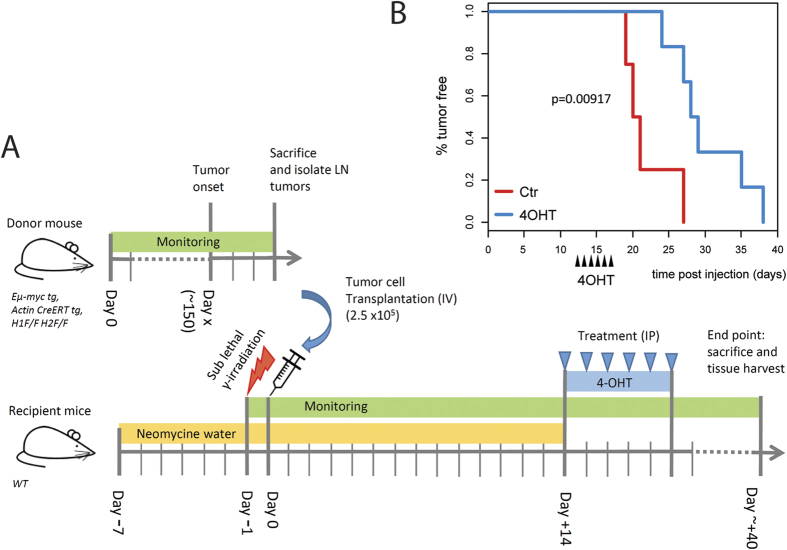
Conditional ablation of Hdac1 and Hdac2 in Eμ-*myc* tumor cells delays tumor appearance *in vivo.* **(A)** Experimental workflow scheme for transplantation experiments. Wild-type syngeneic recipient mice were sub-lethally irradiated (350 cGy of whole-body γ-irradiation) and transplanted intra-venously with lymph node-derived tumor cells from *Hdac1*^***F/F***^*; Hdac2*^***F/F***^; *Actin-creER* tg; Eμ-*myc* tg mice after development of overt malignancy. Recipient mice were treated with neomycin-supplemented drinking water 1 week before transplantation and up to 2 weeks post transplantation. At two weeks post transplantation, conditional KO was induced in one group of mice by intraperitoneal injection of 4-hydroxytamoxifen (4-OHT, 5 × 2 mg). Control mice were injected with vehicle. Mice were monitored for tumor onset and sacrificed when they reached termination criteria (see Material and Methods). **(B)** KPLM tumor-free survival curves of mice transplanted with tumor cells and treated with 4-OHT (n **= **6) or vehicle (Ctr, n **= **4) are shown. Survival is plotted as days post transplantation. The log-rank test was used to determine the level of significance between curves in the two groups.

In order to discriminate between indirect B cell developmental defects and direct effects of *Hdac1* and *Hdac2* ablation, we investigating the role of Hdac1 and Hdac2 in existing tumor cells. Therefore, we performed conditional targeted deletion of *Hdac1* and *Hdac2* in Eμ-*myc* tumor cells using an *in vivo* transplantation approach (Fig. 4A). Briefly, syngeneic recipient mice were injected with Eμ-*myc* lymphoma cells carrying floxed *Hdac1* and *Hdac2* alleles as well as hormone-inducible cre (*Hdac1*^***F/F***^*; Hdac2*^***F/F***^; *Actin-cre* ER tg; Eμ-*myc* tg) and subsequently treated with 4-hydroxytamoxifen (4-OHT) to induce deletion of *Hdac1* and *Hdac2* specifically in transplanted tumor cells. We then performed KPLM tumor-free survival analysis and observed that mice treated with 4-OHT had significantly delayed tumor appearance compared to control mice treated with vehicle (Fig. 4B). Thus, Hdac1 and Hdac2 have a pro-oncogenic role in the Eμ-*myc* dependent tumor progression. This transplantation assay clearly demonstrates that conditional ablation of both *Hdac1* and *Hdac2* directly impacts on existing tumor cells and delays tumor appearance.

### *Hdac1* ^*Δ/Δ*^ *; Hdac2* ^*Δ/*+^ delays Eμ-*myc* tumorigenesis

**Figure 5:**
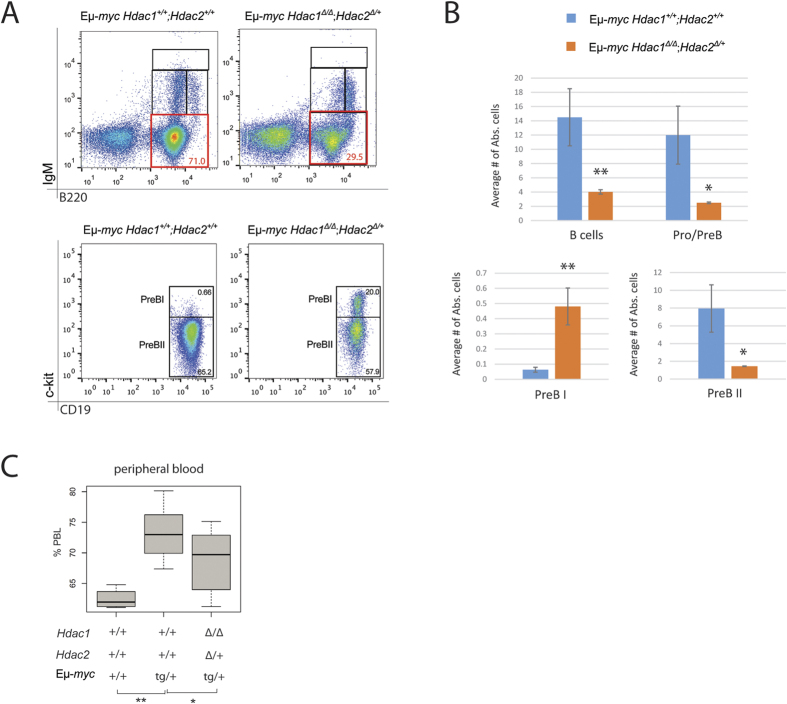
Hdac1^*Δ/Δ*^*; Hdac2*^*Δ/***+**^ Eμ-*myc* mice have delayed tumorigenesis. All experiments were performed in 8-week-old lymphoma-free mice. **(A,B)** BM cells obtained from 8-week old Eμ-*myc* mice with indicated genotypes were stained with B cell surface marker-specific antibodies, including B220, IgM, CD19 and c-kit, and analyzed by flow cytometry (representative dot plots are shown). **(A)** Representative flow cytometry dot plots gated on total BM lymphocytes. Gated regions indicate B cell subsets of interest with frequency in percent. Pro/preB cell subset (B220^+^; IgM^−^) is indicated (red gate, upper panels). Gated PreBI (B220^+^; c-kit^+^; CD19^+^) and PreBII (B220^+^; CD19^+^; c-kit^−^) cell populations are indicated (lower panels). **(B)** Quantification of flow cytometry analysis with s.e.m. (n **= **3 biological replicates). Average percentage of B cells (B220^+^) and Pro/PreB cells (upper plots), and PreBI and PreBII (lower plots). Statistical analysis was performed with Student unpaired 2-tailed *t* test. **(C)** Blood was analyzed with automated blood analyzer. Shown are box plots with frequency (%) of PBL from indicated genotypes (n **= **10). *p*-value calculated with the Wilcoxon Signed-Rank Test. Significant differences are indicated, *p < 0.05; **p < 0.01. N.S., not statistically significant.

As shown above, E*μ*-*myc* tumor development was significantly delayed in mice having a single allele of *Hdac2* and no *Hdac1* (*Hdac1*^***Δ/Δ***^*; Hdac2*^***Δ/*****+**^), while this was not the case in mice with a single allele of *Hdac1* and no *Hdac2* (*Hdac1*^***Δ/*****+**^*; Hdac2*^***Δ/Δ***^; Fig. 2). We analysed 8-week old lymphoma-free Eμ-*myc* mice and first performed flow cytometry analysis of the BM. Interestingly, *Hdac1*^***Δ/Δ***^; *Hdac2*^***Δ/*****+**^; E*μ*-*myc* mice had significantly reduced blasts at Pro/PreB cell stages (Fig. 5A, upper panels) and displayed a strongly reduced number of B cells and Pro/PreB cells in the BM (Fig. 5B, upper panels). Furthermore, we found that these mice had several fold increased PreBI cell numbers and similarly decreased PreBII cell numbers (Fig. 5A, lower panels). Quantification revealed significant changes (Fig. 5B, lower panels). We next measured circulating PBL, and observed that *Hdac1*^***Δ/Δ***^*; Hdac2*^***Δ/*****+**^ Eμ-*myc* mice had significantly lowered PBL counts compared to control Eμ-*myc* mice with normal levels of Hdac1 and Hdac2 (Fig. 5C). Hence, Hdac1^***Δ/Δ***^*; Hdac2*^***Δ/*****+**^ Eμ-*myc* mice have reduced leukemia. Taken together, these results indicate that reduction of Hdac2 in absence of Hdac1 (*Hdac1*^***Δ/Δ***^*; Hdac2*^***Δ/*****+**^) impacts Eμ-*myc* tumorigenesis by reducing the Eμ-*myc-*induced blasts in the BM, resulting in reduced circulating tumor cells and eventually delays Eμ-*myc* tumorigenesis (Fig. 2).

### Hdac1 has a predominant role in non-malignant B cells

**Figure 6:**
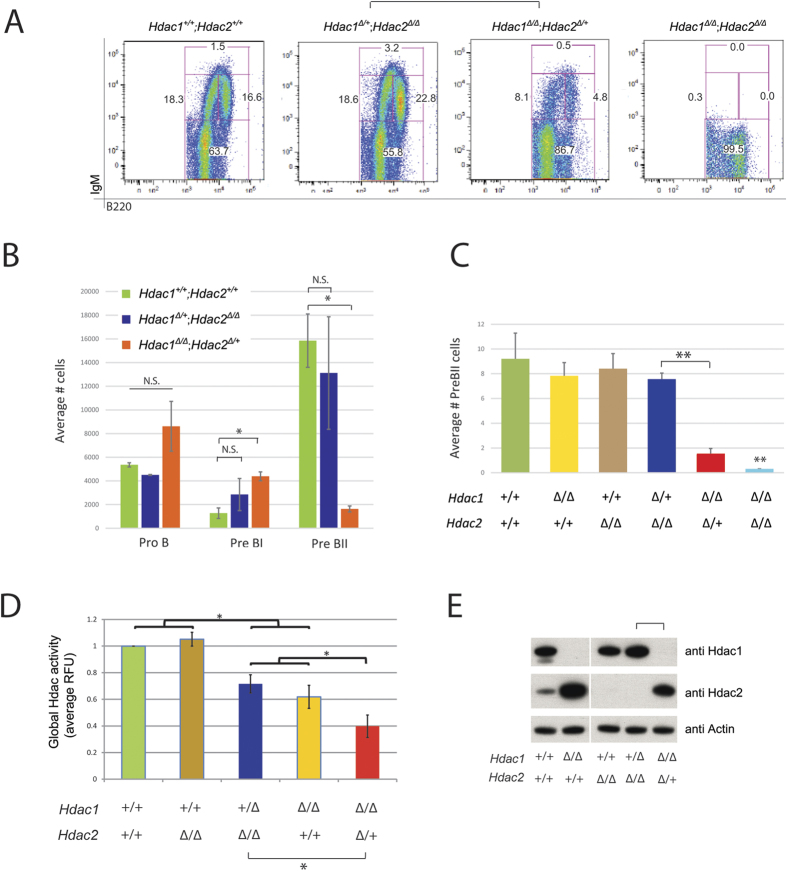
Hdac1 has a predominant role in non-malignant B cells. All experiments were performed in 8-week-old animals. **(A)** Representative flow cytometry dot plots of B220/IgM staining, gated on B220^+^ lymphocytes derived from BM of mice with indicated genotypes. Gated regions in dot plots indicate B cell subsets of interest with frequency in percent: Pro/preB (B220^+^; IgM^−^), ImmatureB (B220^low^; IgM^+^), Transitional B (B220^+^; IgM^+^) and mature B (B220^high^; IgM^+^) cells. **(B)** Quantification of flow cytometry analysis shown in **(A)**. Bar plots represent average numbers of cells (gated 50,000 lymphocytes) from the different B lymphocyte subsets in the BM. **(C)** Quantification of flow cytometry analysis shown in (A). Average numbers of absolute PreBII cells. **(D)** Global Hdac-activity assay performed in CD19^+^ MACS sorted B cells from BM of mice with indicated genotypes. Values are shown in Relative Fluorescence Units (RFU) relative to control *Hdac1*^**+/+**^*; Hdac2*^**+/+**^ cells. **(E)** Immunoblot analysis of Hdac1 and Hdac2 expression, and actin as loading control from CD19^+^ MACS sorted splenic B cells derived from mice with indicated genotypes. The cropped blots originate from a single blot for each protein. The full-length blots are presented in Supplementary Figure 7. All graphs represent mean ± s.e.m. from 3 mice of each genotype. Statistical analysis with Student unpaired 2-tailed *t* test, *p < 0.05; **p < 0.01. N.S., not statistically significant.

In order to test whether the impact of Hdac1 and Hdac2 on B cells is restricted to the Eμ-*myc* background, we examined mice with B cell-specific deletions of different combinations of *Hdac1* and *Hdac2* alleles but no Eμ-*myc* tg (Supplementary Figure 1A). We first examined the effect of *Hdac1* and *Hdac2* ablation in the BM by flow cytometry. We observed that *Hdac1*^***Δ/Δ***^*; Hdac2*^***Δ/*****+**^ but not *Hdac1*^***Δ/*****+**^*; Hdac2*^***Δ/Δ***^ mice had less B cells, whereas *Hdac1*^***Δ/Δ***^*; Hdac2*^***Δ/Δ***^mice had a complete block in B cell development (Fig. 6A). Quantification of the flow cytometry analysis revealed that *Hdac1*^***Δ/Δ***^*; Hdac2*^***Δ/*****+**^, but not *Hdac1*^***Δ/*****+**^*; Hdac2*^***Δ/Δ***^ mice, have higher numbers of PreBI cells and reduced PreBII cell numbers compared to control *Hdac1*^**+/+**^*; Hdac2*^**+/+**^ mice (Fig. 6B). Of note, B cells development was not blocked in *Hdac1*^***Δ/Δ***^*; Hdac2*^***Δ/*****+**^ mice since these mice still had PreBII cells (Fig. 6C) that could develop throughout all B cells stages and eventually fill the complete pool of mature B cells in the spleen (Supplementary Figure 6). In comparison, mice lacking both *Hdac1* and *Hdac2* (*Hdac1*^***Δ/Δ***^*; Hdac2*^***Δ/Δ***^) had almost no PreBII cells (Fig. 6C), as shown previously^30^.

We next determined the effect of *Hdac1* and *Hdac2* ablation on the global Hdac-activity in BM B cells. Interestingly, we observed that progressive ablation of *Hdac1* and *Hdac2* alleles generated a B cell-specific gradient of Hdac-activity, with *Hdac1* having a greater contribution: *Hdac1*^**+/+**^*; Hdac2*^**+/+**^ **= ***Hdac1*^**+/+**^*; Hdac2*^***Δ/Δ***^ **> ***Hdac1*^**+*****/Δ***^*; Hdac2*^***Δ/Δ***^ ≥ *Hdac1*^***Δ/Δ***^*; Hdac2*^**+/+**^ ≥ *Hdac1*^***Δ/Δ***^*; Hdac2*^**+*****/Δ***^ (Fig. 6D). Further assessment by immunoblotting of Hdac1 and Hdac2 protein levels in isolated B cells confirmed that these mice had efficient deletion of Hdac1 and Hdac2 (Fig. 6E). Ablation of *Hdac1* resulted in increased Hdac2 protein levels, as shown previously^30^, while ablation of *Hdac2* did not result in increased Hdac1 proteins levels. These results suggest compensatory regulation of Hdac1 and Hdac2 protein levels in B cells, with a predominant compensation of Hdac2 upon loss of the other paralog (Fig. 6E). Taken together, these data demonstrate that Hdac1 also has a predominant role in non-malignant B cells.

### *Hdac1* ^*Δ/Δ*^ *; Hdac2* ^*Δ/* **+**^ impact on proliferation and apoptosis

**Figure 7:**
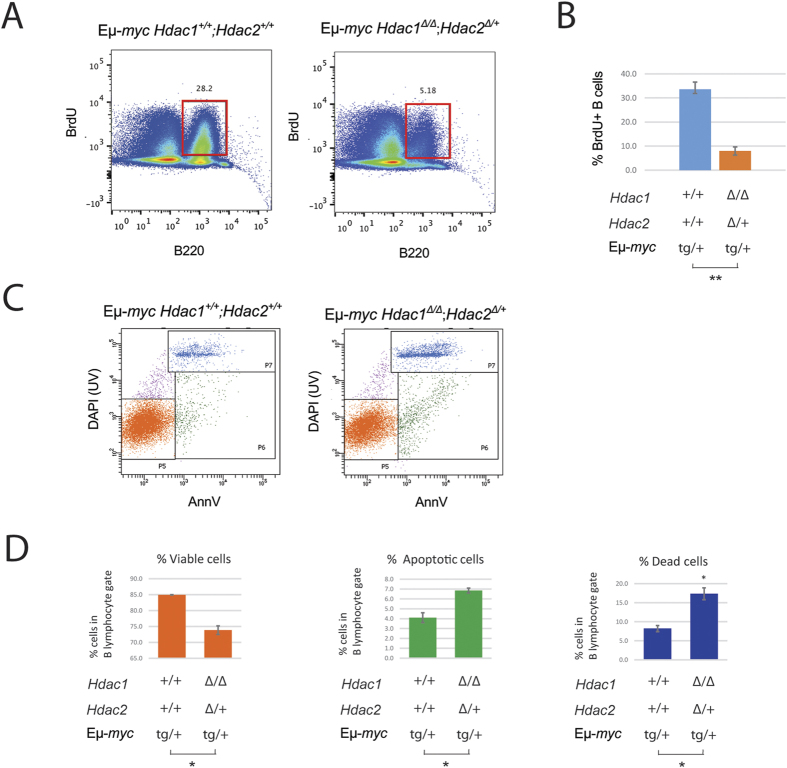
*Hdac1*^*Δ/Δ*^*; Hdac2*^*Δ/***+**^ impact on proliferation and apoptosis. Experiments were performed in 8-week-old lymphoma-free mice. **(A,B)** Proliferation analysis by flow cytometry from BM B cells of mice with indicated genotypes injected with BrdU. **(A)** Representative flow cytometry dot plots from indicated genotypes. Gated regions indicate B220^+^; BrdU^+^ cycling B cells with frequency in percent. **(B)** Quantification from flow cytometry analysis with mean and s.e.m. of BrdU-positive B cells is shown. **(C,D)** BM cells isolated from *Hdac1*^**+/+**^*; Hdac2*^**+/+**^ and *Hdac1*^***Δ/Δ***^*; Hdac2*^***Δ/*****+**^ Eμ-*myc* mice and stained with Annexin V, DAPI and B cell surface markers for flow cytometry analysis. **(C)** Representative flow cytometry dot plots from apoptosis assay: viable B cells (P5; annexinV^−^ DAPI^−^), apoptotic B cells (P6; annexinV^+^ DAPI^−^) cells, and dead cells (P7; annexinV^+^ DAPI^+^). **(D)** Quantification of figure (**C**). Mean percentages and standard deviations are shown. All statistical analysis were performed with the Student unpaired 2-tailed *t* test. Significant differences in means between genotypes are indicated, *p < 0.05.

We previously showed that simultaneous ablation of *Hdac1* and *Hdac2* (*Hdac1*^***Δ/Δ***^*; Hdac2*^***Δ/Δ***^) in non-transformed B cells induced cell cycle arrest and subsequent apoptosis^30^. These findings prompted us to hypothesize that proliferation and apoptosis might also be affected in Eμ-*myc* B cells lacking Hdac1 and Hdac2. Therefore, we first investigated the impact of *Hdac1* and *Hdac2* ablation on proliferation of Eμ-*myc* B cells, by *in vivo* BrdU labelling experiments. We found that *Hdac1*^***Δ/Δ***^*; Hdac2*^***Δ/*****+**^ Eμ-*myc* mice had decreased B cell proliferation, as evidenced by reduced BrdU incorporation (Fig. 7A). Quantification of B220^+^; BrdU^+^ cells revealed a significant decrease (Fig. 7B). We next investigated whether apoptosis was induced in *Hdac1*^***Δ/Δ***^*; Hdac2*^***Δ/*****+**^ Eμ-*myc* B cells, using AnnexinV apoptosis assay by flow cytometry, and indeed observed that *Hdac1*^***Δ/Δ***^*; Hdac2*^***Δ/*****+**^ Eμ-*myc* B cells underwent apoptosis at higher frequencies than control *Hdac1*^**+/+**^*; Hdac2*^**+/+**^ Eμ-*myc* B cells (Fig. 7C). Quantification of these data revealed a significant decrease in viable cells (AnnV^−^;DAPI^−^), concomitant with a significant increase in apoptotic (AnnV^+^;DAPI^−^) and dead cells (AnnV^+^;DAPI^+^; Fig. 7D). In summary, B cells with only one allele of *Hdac2* have decreased proliferation and undergo apoptosis more frequently. Taken together, our data demonstrate that *Hdac1*^***Δ/Δ***^*; Hdac2*^***Δ/*****+**^ reduces Eμ-*myc* tumorigenesis by decreasing proliferation and inducing apoptosis.

## Discussion

In this study, we used targeted conditional deletion of *Hdac1* and *Hdac2*, to investigate the functional role of these enzymes in the *Eμ-myc* murine B cell lymphoma model. Our data reveal a predominant role of *Hdac1* in both *Eμ-myc* tg B cells and non-malignant B cells. We demonstrate that *Hdac1* and *Hdac2* have a gene dose-dependent pro-oncogenic role in *Eμ-myc* tumorigenesis with a predominant role of *Hdac1*. Our results highlight the tumor-promoting role of Hdac1 and Hdac2 in both *Eμ-myc* tumorigenesis and tumor maintenance.

In accordance with our previous study with young B cell-specific *Hdac1* and/or *Hdac2* KO mice^30^, we show here that Hdac1 and Hdac2 do not have a tumor suppressor function in B lymphocytes of old mice (Fig. 1). Indeed, we found that ablation of *Hdac1* and/or *Hdac2* in non-malignant B cells did not lead to spontaneous tumor development, in contrast to T cells^32^,^33^, and epidermal cells^34^, in which Hdac1 and Hdac2 were reported to act as tumor suppressors. One plausible interpretation of this apparent discrepancy between our data and these previous studies could be a cell type-specific role of Hdac1 and Hdac2.

We further investigated the function of Hdac1 and Hdac2 in the *Eμ-myc* murine B cell lymphoma model. In accordance with previous reports using HDACis in B lymphoid cancer models^9^,^10^,^11^, we demonstrated using genetic ablation that Hdac1 and Hdac2 have pro-oncogenic roles during Eμ-*myc* tumorigenesis (Fig. 2). Interestingly, these findings differ from previous studies using a skin tumor model^34^, or APL^24^, in which Hdac1 (but not Hdac2) was reported to act as a tumor suppressor during tumorigenesis. This divergence supports the idea that tumor type- or oncogene-specific effects may be decisive. Moreover, we previously observed that Hdac1 and Hdac2 have partly different target preferences^30^, suggesting that they might regulate different set of genes in a cell type-specific manner.

We further show that complete deletion of both *Hdac1* and *Hdac2* (*Hdac1*^***Δ/Δ***^*; Hdac2*^***Δ/Δ***^) prevents Eμ-*myc* tumorigenesis, whereas ablation of either *Hdac1* (*Hdac1*^***Δ/Δ***^*; Hdac2*^**+/+**^) or *Hdac2* (*Hdac1*^**+/+**^*; Hdac2*^***Δ/Δ***^) had no effect (Fig. 2). In line with this, we found that absence of these two *Hdacs* prevents Eμ-*myc* splenomegaly, HG-NHL occurrence, reduces leukemia, and stops B cell blasts accumulation, which otherwise dominates the BM of Eμ-*myc* mice (Fig. 3). Thus, ablation of *Hdac1* and *Hdac2* prevents tumorigenesis already in the BM by preventing Eμ-*myc-*induced blasts at early B cell stage. This is in agreement with our earlier report in non-transformed B cells, where ablation of *Hdac1* and *Hdac2* (*Hdac1*^*Δ/Δ*^*; Hdac2*^*Δ/Δ*^) using *Mb1-cre* induced a cell cycle block and apoptosis at the PreBII cell stage^30^. However, the non-inducible *Mb1-Cre* system does not allow proper discrimination between indirect B cell developmental defects and direct effects of *Hdac1* and *Hdac2* ablation in *Hdac1*^*Δ/Δ*^*; Hdac2*^*Δ/Δ*^ Eμ-*myc* mice. We therefore used two different approaches to investigate the direct effect of Hdac1 and Hdac2: *i)* the use of mice with only one allele of *Hdac2* (*Hdac1*^*Δ/Δ*^*; Hdac2*^*Δ/***+**^), which do not have this block in B cell development to study the tumorigenesis and *ii)* a transplantation approach allowing conditional deletion of *Hdac1* and *Hdac2* (using inducible *CreERT*) in existing tumor cells and test the effect of *Hdac1* and *Hdac2* ablation on tumor maintenance. Our transplantation experiment (Fig. 4) clearly demonstrates that conditional ablation of both *Hdac1* and *Hdac2* has a direct impact on existing tumor cells, since we observed significantly delayed tumor appearance. Interestingly, we observed that *Hdac1* and *Hdac2* are not deleted in tumors arising in 4-OHT treated transplanted mice (Supplementary Figure 5), suggesting that the delayed tumor growth observed represents cells that have escaped deletion of *Hdac1* and *Hdac2*. From these findings we conclude that the partial effect we observed after 4-OHT treatment of transplanted recipient mice (Fig. 4) can be explained by incomplete elimination of *Hdac1* and *Hdac2* using the tamoxifen inducible CreERT system. Our findings demonstrate that loss of *Hdac1* and *Hdac2* has a direct impact on Eμ-*myc* tg B cells and demonstrate a critical pro-oncogenic role of Hdac1 and Hdac2 in Eμ-*myc* tumor progression.

Eμ-*myc* mice with a single allele of *Hdac2* and no *Hdac1* (*Hdac1*^***Δ/Δ***^*; Hdac2*^***Δ/*****+**^), but not with a single allele of *Hdac1* in absence of *Hdac2* (*Hdac1*^**+*****/Δ***^*; Hdac2*^***Δ/Δ***^), exhibited delayed tumor development (Fig. 2). This demonstrates that Eμ-*myc* tumorigenesis is *Hdac1* and *Hdac2* gene dose-dependent, and identifies a predominant role of *Hdac1*. We further observed that Hdac1 has a predominant role also in non-malignant B cells (Fig. 6). *Hdac1*^***Δ/Δ***^*; Hdac2*^***Δ/*****+**^ mice, but not *Hdac1*^**+*****/Δ***^*; Hdac2*^***Δ/Δ***^ mice, had a reduction in PreBII cell numbers (Fig. 6C). Furthermore, ablation of Hdac1 resulted in a strong increase of Hdac2 protein levels, as previously observed^30^. Interestingly, this increase in Hdac2 levels was not sufficient to compensate for the absence of Hdac1, indicating partially redundant functions and highlighting the predominant role of Hdac1. Furthermore, we observed that *Hdac1*^***Δ/Δ***^*; Hdac2*^***Δ/*****+**^ B cells had significantly reduced Hdac activity compared to *Hdac1*^**+*****/Δ***^*; Hdac2*^***Δ/Δ***^ B cells. These data demonstrate the predominant role of Hdac1, and suggest that a critical level of Hdac activity may be required for Eμ-*myc* tumorigenesis.

Similar to *Hdac1*^***Δ/Δ***^*; Hdac2*^***Δ/Δ***^, we found that *Hdac1*^***Δ/Δ***^*; Hdac2*^***Δ/*****+**^ impacted Eμ-*myc* tumorigenesis by reducing the Eμ-*myc-*induced blasts at the early preBII cell stage in the BM, resulting in reduced circulating tumor cells (Fig. 5). We further investigated whether the impact of *Hdac1*^*Δ/Δ*^*; Hdac2*^*Δ/***+**^ in Eμ-*myc* B cells could be due to proliferation defects and/or apoptosis. Strikingly, we found that *Hdac1*^***Δ/Δ***^*; Hdac2*^***Δ/*****+**^ decreased proliferation and increased apoptosis in Eμ-*myc* B cells (Fig. 7). Taken together, these findings demonstrate that *Hdac1*^*Δ/Δ*^*; Hdac2*^*Δ/***+**^ reduces Eμ-*myc-*induced blasts in the BM and delays tumorigenesis by decreasing proliferation and inducing apoptosis. Hence, we conclude that Hdac1 and Hdac2 have pro-oncogenic roles in Eμ-*myc* tumorigenesis. These findings are consistent with earlier studies reporting that complete loss of Hdac1 and Hdac2 induces cell death in proliferating cells, including B and T cells^30^,^32^,^33^. In line with this, Hdac1 and Hdac2 were shown to be the major targets for HDACi-mediated apoptosis induction in leukemic cell lines^40^. Furthermore, a recent study also showed that ablation of both Hdac1 and Hdac2 decreases proliferation and induces apoptosis in Eμ-*myc* tumor cells^23^. Hence, an effect on proliferation and apoptosis upon Hdac1 and Hdac2 ablation could likely explain the delayed tumor appearance in our transplantation experiment (Fig. 4).

Our results describe the pro-oncogenic roles of Hdac1 and Hdac2 in Eμ*-myc* tumorigenesis and tumor maintenance and support the clinical use of HDACis. Previous studies clearly demonstrated the therapeutic efficacy of pan-HDACis in Eμ-*myc* lymphomas^41^,^42^,^43^,^44^,^45^. Interestingly, we found that human HDAC1, but not human HDAC2, mRNA expression is increased in some Burkitt’s lymphoma (BL) and diffuse large B cell lymphoma (DLBCL) cancer cell lines and human lymphoma samples, when compared to other cancer cell lines or other human cancer samples, respectively (Supplementary Figure 3). Hence, these data are consistent with our findings outlined above, revealing a predominant role of Hdac1. Our findings demonstrate that Hdac1 can be considered an important factor in Eμ*-myc* tumorigenesis, and suggest that selective HDAC1 (and HDAC2) inhibitors could be effective for the treatment of BL, as modeled by our preclinical Eμ-*myc* system, and possibly other hematological malignancies, including some DLBCL. Accordingly, several HDAC1 and HDAC2 isoform-selective inhibitors were recently shown to have *in vitro* and/or *in vivo* therapeutic efficacy in pre-clinical models such as Eμ-*myc*^23^,^42^.

In conclusion, our results demonstrate that Hdac1 and Hdac2 promote tumor initiation and progression in Eμ-myc mice and that they impact on proliferation and apoptosis. To the best of our knowledge, this is the first study showing a gene dose-dependent pro-oncogenic role of *Hdac1* and *Hdac2* in tumorigenesis, with a predominant role of *Hdac1*. Future research will focus on elucidating the underlying molecular mechanisms by which Hdac1 and Hdac2 regulate proliferation and apoptosis in malignant and non-malignant cells. Our study raises the prospect of using selective HDAC1 and HDAC2 inhibitors for the treatment of BL and other B cell lymphomas with Myc deregulation, with possibly less side effects than pan-HDACis currently used.

## Material and Methods

### Experimental mice

All experiments were performed in accordance with Swiss federal guidelines for animal experimentation (Art.13a TSchG; Art. 60–62 TSchV) and approved by the FMI Animal committee and the local veterinary authorities (Kantonales Veterinäramt of Kanton Basel-Stadt, permit no. 2384–03). All efforts were made to minimize animal suffering and to reduce the number of animal used.

*Hdac1*^***F/F***^*; Hdac2*^***F/F***^ conditional knockout (KO) mice have been previously described and characterized^30^. B lymphocyte-specific deletion of *Hdac1* and/or *Hdac2* was obtained by crossing *Hdac1*^***F/F***^ *and Hdac2*^***F/F***^ mice with heterozygote *Mb1-cre* transgenic (tg) mice^30^. For transplantation, *Actin-Cre* ER mice (The Jackson Laboratory; B6.Cg-Tg(CAG-cre/Esr1)5Amc/J) were used to conditionally delete *Hdac1* and *Hdac2* using tamoxifen. *Hdac1* and *Hdac2* conditional KO mice were interbred to congenic C57BL/6 heterozygote Eμ-*myc* tg mice (The Jackson Laboratory; B6.Cg-Tg (IghMyc)22Bri/J)^35^. All mice were in C57BL/6 genetic backgrounds (backcrossing at least 11 generations). The mice were housed in groups of one to five at 25 °C with a 12:12 h light-dark cycle and received a standard laboratory diet containing 0.8% phosphorus and 1.1% calcium (NAFAG 890, Kliba, Basel, Switzerland) and water *ad libitum*.

### Genotyping PCR

Polymerase chain reaction (PCR)–based genotyping was performed on tail-derived DNA. Mice were genotyped for *Hdac1* and *Hdac2* conditional alleles as described previously^30^. The following primer sets were used: *Hdac1* flox or WT (forward; 5′-CCTGTGTCATTAGAATCTACTT, and reverse; 5′-GGTAGTTCACAGCATAGTACTT); *Hdac1* KO (forward; 5′-GTTACGTCAATGACATCGTCCT, and reverse; 5′-GGTAGTTCACAGCATAGTACTT); *Hdac2* flox or WT (forward; 5′-CCCTTTAGGTGTGAGTACAT, and reverse; 5′-rev: AACCTGGAGAGGACAGCAAA); *Hdac2* KO (forward; 5′-CCACAGGGAAAAGGAAACAA, and reverse; 5′-AACCTGGAGAGGACAGCAAA). Eμ-*myc* tg (forward; 5′-TCCAGGGTACATGGCGTATT; and reverse; 5′-TCGGCTGAACTGTGTTCTTG), based on previously published insertion site of *c-myc*^46^. *Mb1-cre* tg (forward; 5′-GGGAAGAAAGAGGCCATAGG; and reverse, 5′-TCCCTCACATCCTCAGGTTC). *Actin-Cre* tg (forward; 5′-GCGGTCTGGCAGTAAAAACTATC; and reverse, 5′-CAGAGACGGAAATCCATCGCTC). PCRs were performed using the GoTaq Flexi DNA Polymerase Kit (Promega, Cat.M8306) and MJ Mini Thermal Cyclers (BioRad).

### RNA isolation and qRT–PCR

Total RNA was isolated using an RNeasy Mini kit (Qiagen) followed by cDNA synthesis using Improm Reverse Transcriptase (RT) Kit (Promega) according to the manufacturer’s protocol. 5–10 ng of cDNA were used to perform Semiquantitative real-time PCR using MESA GREEN qPCR MasterMix Plus for SYBR Assay (Eurogentec) on an ABI PRISM7000 Sequence Detection System (Applied Biosytems). Each reported value is an average of three independent experiments. Relative expression levels were determined by normalizing to *gapdh* expression using the ΔΔC_t_ method. The following primers were used: for *c-myc* (forward; 5′-TTTGTCTATTTGGGGACAGTGTT; and reverse; 5′-CATCGTCGTGGCTGTCTG); for *Gapdh* (forward; 5′-GCCTCGTCCCGTAGACAAAAT; and reverse; 5′-TTCCCATTCTCGGCCTTGA).

### Kaplan-Meyer (KPLM) tumor-free survival analysis

Tumor development was monitored every 2–3 days by palpation of cervical, axillary, and inguinal regions for characteristic “water wing” appearance described for Eμ-*myc* tg mice^37^. Typically, moribund mice presented with several of the following visible features: enlarged lymph nodes, hunched posture, dyspnea, weight loss, ruffled coats, paralysis, and immobility. Mice were monitored over a period of 300 days for tumor onset and sacrificed when moribund, or reaching tumor-specific endpoints (lymph nodes>1 cm). For moribund mice without tumors, the date of euthanasia was used as the date of death in survival studies. Tumors were isolated, weighted, and prepared for histopathology or protein and RNA extraction. The survival rate was calculated using the Kaplan-Meier method, using R (R Project for Statistical Computing).

### Blood sampling and analysis

Mice were bled at 4 and 8 weeks and blood was collected in EDTA pre-coated tubes. Samples were analyzed utilizing a fully automated hematology analyzer (Sysmex XT-2000i).

### Histopathological analysis

Biopsies were formalin-fixed (Shandon Formal-Fixx, Thermo Scientific) for 24 h, dehydrated, paraffin-embedded, cut into 3-μm-thick sections, and stained with hematoxylin and eosin (Merck). Pathological analysis was performed according to the Bethesda proposals for classification of lymphoid neoplasms in mice^38^.

### Cell preparation

Single cell suspensions were prepared from BMs by flushing tibia and femur with PBS supplemented with 3% fetal bovine serum. Single-cell suspensions from spleen and lymph nodes were prepared by squeezing splenocytes and lymphocytes from their capsule through a 40-μm nylon mesh of the cell strainer. Peripheral blood was obtained by venous puncture or at autopsy by cardiac puncture. Red blood cells were depleted by lysis in Gey’s solution prior to staining.

### Immunofluorescent staining and flow cytometric analysis

Flow cytometry was done according to standard procedures^30^. The following directly conjugated antibodies were used: anti-CD45R/B220-FITC (clone RA3-6B2), anti-CD25-PE (clone PC61), anti-IgM-APC (clone II/41), anti-IgD-BV605 (clone 11–26 c.2a), were all purchase from BD Biosciences; anti-CD117/c-kit-APC (clone 2B8, eBioscience); and anti-CD19-PE-Cy7 (clone 6D5, Biolegend). All flow cytometry analyses were performed using a multicolor BD LSRII Flow Cytometer (Becton Dickinson). Data were analyzed using Flow-Jo (Tree Star) software.

### B cell isolation by magnetic-activated cell sorting (MACS)

Separation of B cells was performed using positive selection with CD19 monoclonal antibodies coupled to magnetic microbeads (anti-CD19 MicroBeads) according to the manufacturer’s protocol (MACS, Miltenyi Biotec). Control flow cytometry from MACS separated cells revealed 95% purity.

### *In vivo* cell cycle analysis by bromodeoxyuridine (BrdU) incorporation

For *in vivo* BrdU incorporation, mice were injected intraperitoneally with 1.5 mg of BrdU solution (10 mg/mL; BD Bioscience) and sacrificed 24 hours later. BrdU staining was performed according to the manufacturer’s protocol (BD Bioscience). BM cells were analyzed by flow cytometry. Percentages of cells in G_0_/G_1_-, S-, and G_2_/M-phases of the cell cycle were determined by manual gating.

### Apoptosis assay

Apoptotic lymphocytes were determined using an antibody against AnnexinV conjugated to FITC (AnnexinV apoptosis detection kit, BD Biosciences) and *DAPI* (Sigma) counterstain, following the manufacturer’s protocol. Percentages of apoptotic cell (FITC^+^, DAPI^−^) were determined by manual gating.

### Protein extracts and Western blot analysis

Cells were harvested in cold RIPA buffer containing 50 mM Tris, 150 mM sodium chloride, 1% Nonidet P-40, 0.25% sodium deoxycholate, 1 mM EDTA, 0.1% SDS and protease inhibitors (Roche). Protein concentrations were determined by Bradford assay, and equal amounts of protein (20 ug) were loaded on 4–12% NuPAGE Bis-Tris Mini Gels (life technologies) separated on SDS–PAGE followed by transfer onto a PVDF transfer membrane (Immobilon-P, Milipore). The following antibodies were used: anti-mouse actin (ab5, Neo Markers), anti-mouse Hdac1 and anti-mouse Hdac2 (provided by Dr. Christian Seiser, Biocenter, Vienna)^47^. Antibodies were diluted 1:1,000.

### *In vitro* Hdac-activity assay

Global Hdac activity was measured with the *Fluor-de-Lys* Hdac assay kit (Enzo; BML-KI104-0050) according to the manufacturer’s protocol. Fluorescence intensity was detected with Spectromax Gemini plate reader (Molecular Devices).

### Eμ-*myc* lymphoma transplantation

Syngeneic C57BL/6 recipient mice (The Jackson Laboratory; B6.SJL-Ptprca Pepcb/BoyJ) were sub-lethally irradiated (350 cGy whole-body γ-irradiation) using a Rad-source RS2000 irradiator (1.2 Gy/min) and transplanted intra-venously with 2.5 × 10^5^ thawed cryopreserved lymph node-derived tumor cells from *Hdac1*^***F/F***^*; Hdac2*^***F/F***^, *Actin-cre*ER tg, Eμ-*myc* tg donor mice, as previously described^36^,^48^. Recipient mice were treated with neomycin-supplemented drinking water (2 mg/ml; sigma) 1 week before, and 2 weeks post transplantation. 14 days post transplantation, conditional KO was induced by intraperitoneal injection of 4-hydroxytamoxifen (4-OHT; 5 × 2 mg). Control mice were injected with vehicle control (ethanol). Mice were monitored for tumor onset and sacrificed when they reached termination criteria as described above.

### Oncomine and CCLE database analysis

Human *Hdac1* (*HDAC1*) and human *Hdac2* (*HDAC2*) mRNA expression levels were compared in human tumor samples and human cancer cell lines using the publicly available databases Oncomine (http://www.oncomine.org) and Cancer Cell Line Encyclopedia (CCLE), respectively^49^,^50^,^51^.

### Statistical analysis

Data are represented as mean ± s.e.m. (standart error measurement of mean). For all analyses, several independent experiments (N ≥ 3) were carried out. Student’s unpaired 2-tailed *t-*tests were performed for all analyses using Microsoft Excel. Statistical significance was determined by p values: N.S. *p* > 0.05, *p < 0.05, **p < 0.01. Statistical analysis of the KPLM survival curves was done using the log-rank test in R (www.r-project.org).

## Additional Information

**How to cite this article**: Pillonel, V. *et al*. Histone deacetylase 1 plays a predominant pro-oncogenic role in Eµ-*myc* driven B cell lymphoma. *Sci. Rep.* **6**, 37772; doi: 10.1038/srep37772 (2016).

**Publisher's note:** Springer Nature remains neutral with regard to jurisdictional claims in published maps and institutional affiliations.

## Supplementary Material

Supplementary Material

## References

[b1] Reichert N., Choukrallah M. A. & Matthias P. Multiple roles of class I HDACs in proliferation, differentiation, and development. Cellular and molecular life sciences: CMLS 69, 2173–2187, doi: 10.1007/s00018-012-0921-9 (2012). 22286122 PMC11115120

[b2] Dawson M. A. & Kouzarides T. Cancer epigenetics: from mechanism to therapy. Cell 150, 12–27, doi: 10.1016/j.cell.2012.06.013 (2012). 22770212

[b3] Jenuwein T. & Allis C. D. Translating the histone code. Science (New York, N.Y.) 293, 1074–1080, doi: 10.1126/science.1063127 (2001). 11498575

[b4] Choudhary C. . Lysine acetylation targets protein complexes and co-regulates major cellular functions. Science (New York, N.Y.) 325, 834–840, doi: 10.1126/science.1175371 (2009). 19608861

[b5] Glozak M. A., Sengupta N., Zhang X. & Seto E. Acetylation and deacetylation of non-histone proteins. Gene 363, 15–23, doi: 10.1016/j.gene.2005.09.010 (2005). 16289629

[b6] Olzscha H., Sheikh S. & La Thangue N. B. Deacetylation of chromatin and gene expression regulation: a new target for epigenetic therapy. Critical reviews in oncogenesis 20, 1–17 (2015). 25746101 10.1615/critrevoncog.2014012463

[b7] Mercurio C., Minucci S. & Pelicci P. G. Histone deacetylases and epigenetic therapies of hematological malignancies. Pharmacological research 62, 18–34, doi: 10.1016/j.phrs.2010.02.010 (2010). 20219679

[b8] Hagelkruys A., Sawicka A., Rennmayr M. & Seiser C. The biology of HDAC in cancer: the nuclear and epigenetic components. Handbook of experimental pharmacology 206, 13–37, doi: 10.1007/978-3-642-21631-2_2 (2011). 21879444

[b9] Haery L., Thompson R. C. & Gilmore T. D. Histone acetyltransferases and histone deacetylases in B- and T-cell development, physiology and malignancy. Genes & cancer 6, 184–213 (2015). 26124919 10.18632/genesandcancer.65 PMC4482241

[b10] Falkenberg K. J. & Johnstone R. W. Histone deacetylases and their inhibitors in cancer, neurological diseases and immune disorders. Nat Rev Drug Discov 13, 673–691, doi: 10.1038/nrd4360 (2014). 25131830

[b11] West A. C. & Johnstone R. W. New and emerging HDAC inhibitors for cancer treatment. The Journal of clinical investigation 124, 30–39, doi: 10.1172/jci69738 (2014). 24382387 PMC3871231

[b12] Bantscheff M. . Chemoproteomics profiling of HDAC inhibitors reveals selective targeting of HDAC complexes. Nat Biotech 29, 255–265 (2011). 10.1038/nbt.1759 21258344

[b13] Ghobrial I. M. . Results of a phase 2 trial of the single-agent histone deacetylase inhibitor panobinostat in patients with relapsed/refractory Waldenstrom macroglobulinemia. Blood 121, 1296–1303, doi: 10.1182/blood-2012-06-439307 (2013). 23287861 PMC3578951

[b14] Richardson P. G. . PANORAMA 2: panobinostat in combination with bortezomib and dexamethasone in patients with relapsed and bortezomib-refractory myeloma. Blood 122, 2331–2337, doi: 10.1182/blood-2013-01-481325 (2013). 23950178

[b15] San-Miguel J. F. . Phase Ib study of panobinostat and bortezomib in relapsed or relapsed and refractory multiple myeloma. Journal of clinical oncology: official journal of the American Society of Clinical Oncology 31, 3696–3703, doi: 10.1200/JCO.2012.46.7068 (2013). 24019544

[b16] Rasheed W., Bishton M., Johnstone R. W. & Prince H. M. Histone deacetylase inhibitors in lymphoma and solid malignancies. Expert review of anticancer therapy 8, 413–432, doi: 10.1586/14737140.8.3.413 (2008). 18366289

[b17] Ononye S. N., van Heyst M., Falcone E. M., Anderson A. C. & Wright D. L. Toward isozyme-selective inhibitors of histone deacetylase as therapeutic agents for the treatment of cancer. Pharmaceutical patent analyst 1, 207–221, doi: 10.4155/ppa.12.21 (2012). 24163736 PMC3807748

[b18] Glaser K. B. . Role of class I and class II histone deacetylases in carcinoma cells using siRNA. Biochemical and biophysical research communications 310, 529–536 (2003). 14521942 10.1016/j.bbrc.2003.09.043

[b19] Haberland M., Johnson A., Mokalled M. H., Montgomery R. L. & Olson E. N. Genetic dissection of histone deacetylase requirement in tumor cells. Proceedings of the National Academy of Sciences of the United States of America 106, 7751–7755, doi: 10.1073/pnas.0903139106 (2009). 19416910 PMC2683118

[b20] Mottet D. . HDAC4 represses p21(WAF1/Cip1) expression in human cancer cells through a Sp1-dependent, p53-independent mechanism. Oncogene 28, 243–256, doi: 10.1038/onc.2008.371 (2009). 18850004

[b21] Senese S. . Role for histone deacetylase 1 in human tumor cell proliferation. Molecular and cellular biology 27, 4784–4795, doi: 10.1128/mcb.00494-07 (2007). 17470557 PMC1951481

[b22] Wilson A. J. . HDAC4 promotes growth of colon cancer cells via repression of p21. Molecular biology of the cell 19, 4062–4075, doi: 10.1091/mbc.E08-02-0139 (2008). 18632985 PMC2555950

[b23] Matthews G. M. . Functional-genetic dissection of HDAC dependencies in mouse lymphoid and myeloid malignancies. Blood 126, 2392–2403, doi: 10.1182/blood-2015-03-632984 (2015). 26447190 PMC4653767

[b24] Santoro F. . A dual role for Hdac1: oncosuppressor in tumorigenesis, oncogene in tumor maintenance. Blood 121, 3459–3468, doi: 10.1182/blood-2012-10-461988 (2013). 23440245

[b25] Montgomery R. L. . Histone deacetylases 1 and 2 redundantly regulate cardiac morphogenesis, growth, and contractility. Genes & development 21, 1790–1802, doi: 10.1101/gad.1563807 (2007). 17639084 PMC1920173

[b26] LeBoeuf M. . Hdac1 and Hdac2 act redundantly to control p63 and p53 functions in epidermal progenitor cells. Developmental cell 19, 807–818, doi: 10.1016/j.devcel.2010.10.015 (2010). 21093383 PMC3003338

[b27] Haberland M., Carrer M., Mokalled M. H., Montgomery R. L. & Olson E. N. Redundant control of adipogenesis by histone deacetylases 1 and 2. The Journal of biological chemistry 285, 14663–14670, doi: 10.1074/jbc.M109.081679 (2010). 20190228 PMC2863240

[b28] Ma P., Pan H., Montgomery R. L., Olson E. N. & Schultz R. M. Compensatory functions of histone deacetylase 1 (HDAC1) and HDAC2 regulate transcription and apoptosis during mouse oocyte development. Proceedings of the National Academy of Sciences of the United States of America 109, E481–489, doi: 10.1073/pnas.1118403109 (2012). 22223663 PMC3286984

[b29] Montgomery R. L., Hsieh J., Barbosa A. C., Richardson J. A. & Olson E. N. Histone deacetylases 1 and 2 control the progression of neural precursors to neurons during brain development. Proceedings of the National Academy of Sciences of the United States of America 106, 7876–7881, doi: 10.1073/pnas.0902750106 (2009). 19380719 PMC2683090

[b30] Yamaguchi T. . Histone deacetylases 1 and 2 act in concert to promote the G1-to-S progression. Genes & development 24, 455–469, doi: 10.1101/gad.552310 (2010). 20194438 PMC2827841

[b31] Wilting R. H. . Overlapping functions of Hdac1 and Hdac2 in cell cycle regulation and haematopoiesis. The EMBO journal 29, 2586–2597, doi: 10.1038/emboj.2010.136 (2010). 20571512 PMC2928690

[b32] Dovey O. M. . Histone deacetylase 1 and 2 are essential for normal T-cell development and genomic stability in mice. Blood 121, 1335–1344, doi: 10.1182/blood-2012-07-441949 (2013). 23287868 PMC3836254

[b33] Heideman M. R. . Dosage-dependent tumor suppression by histone deacetylases 1 and 2 through regulation of c-Myc collaborating genes and p53 function. Blood 121, 2038–2050, doi: 10.1182/blood-2012-08-450916 (2013). 23327920 PMC3596963

[b34] Winter M. . Divergent roles of HDAC1 and HDAC2 in the regulation of epidermal development and tumorigenesis. The EMBO journal 32, 3176–3191, doi: 10.1038/emboj.2013.243 (2013). 24240174 PMC3981143

[b35] Adams J. M. . The c-myc oncogene driven by immunoglobulin enhancers induces lymphoid malignancy in transgenic mice. Nature 318, 533–538 (1985). 3906410 10.1038/318533a0

[b36] Langdon W. Y., Harris A. W., Cory S. & Adams J. M. The c-myc oncogene perturbs B lymphocyte development in E-mu-myc transgenic mice. Cell 47, 11–18 (1986). 3093082 10.1016/0092-8674(86)90361-2

[b37] Harris A. W. . The E mu-myc transgenic mouse. A model for high-incidence spontaneous lymphoma and leukemia of early B cells. The Journal of experimental medicine 167, 353–371 (1988). 3258007 10.1084/jem.167.2.353 PMC2188841

[b38] Morse H. C. 3rd. . Bethesda proposals for classification of lymphoid neoplasms in mice. Blood 100, 246–258 (2002). 12070034 10.1182/blood.v100.1.246

[b39] Sidman C. L., Shaffer D. J., Jacobsen K., Vargas S. R. & Osmond D. G. Cell populations during tumorigenesis in Eu-myc transgenic mice. Leukemia 7, 887–895 (1993). 8501983

[b40] Inoue S., Mai A., Dyer M. J. & Cohen G. M. Inhibition of histone deacetylase class I but not class II is critical for the sensitization of leukemic cells to tumor necrosis factor-related apoptosis-inducing ligand-induced apoptosis. Cancer research 66, 6785–6792, doi: 10.1158/0008-5472.can-05-4563 (2006). 16818655

[b41] Lindemann R. K. . Analysis of the apoptotic and therapeutic activities of histone deacetylase inhibitors by using a mouse model of B cell lymphoma. Proceedings of the National Academy of Sciences of the United States of America 104, 8071–8076, doi: 10.1073/pnas.0702294104 (2007). 17470784 PMC1876573

[b42] Newbold A. . Molecular and biologic analysis of histone deacetylase inhibitors with diverse specificities. Molecular cancer therapeutics 12, 2709–2721, doi: 10.1158/1535-7163.mct-13-0626 (2013). 24092806

[b43] Newbold A., Salmon J. M., Martin B. P., Stanley K. & Johnstone R. W. The role of p21(waf1/cip1) and p27(Kip1) in HDACi-mediated tumor cell death and cell cycle arrest in the Emu-myc model of B-cell lymphoma. Oncogene 33, 5415–5423, doi: 10.1038/onc.2013.482 (2014). 24292681

[b44] Newbold A. . Characterisation of the novel apoptotic and therapeutic activities of the histone deacetylase inhibitor romidepsin. Molecular cancer therapeutics 7, 1066–1079, doi: 10.1158/1535-7163.mct-07-2256 (2008). 18483296

[b45] Ellis L. . The histone deacetylase inhibitors LAQ824 and LBH589 do not require death receptor signaling or a functional apoptosome to mediate tumor cell death or therapeutic efficacy. Blood 114, 380–393, doi: 10.1182/blood-2008-10-182758 (2009). 19383971 PMC4580966

[b46] Corcoran L. M., Cory S. & Adams J. M. Transposition of the immunoglobulin heavy chain enhancer to the myc oncogene in a murine plasmacytoma. Cell 40, 71–79 (1985). 2981633 10.1016/0092-8674(85)90310-1

[b47] Zupkovitz G. . Negative and positive regulation of gene expression by mouse histone deacetylase 1. Molecular and cellular biology 26, 7913–7928, doi: 10.1128/mcb.01220-06 (2006). 16940178 PMC1636735

[b48] Wall M. . The mTORC1 inhibitor everolimus prevents and treats Emu-Myc lymphoma by restoring oncogene-induced senescence. Cancer discovery 3, 82–95, doi: 10.1158/2159-8290.cd-12-0404 (2013). 23242809 PMC3547521

[b49] Rhodes D. R. . Oncomine 3.0: genes, pathways, and networks in a collection of 18,000 cancer gene expression profiles. Neoplasia (New York, N.Y.) 9, 166–180 (2007). 10.1593/neo.07112 PMC1813932 17356713

[b50] Rhodes D. R. . ONCOMINE: a cancer microarray database and integrated data-mining platform. Neoplasia (New York, N.Y.) 6, 1–6 (2004). 10.1016/s1476-5586(04)80047-2 PMC1635162 15068665

[b51] Barretina J. . The Cancer Cell Line Encyclopedia enables predictive modelling of anticancer drug sensitivity. Nature 483, 603–607, doi: 10.1038/nature11003 (2012). 22460905 PMC3320027

