# Supplementary material for: Histone deacetylase 1 plays a predominant pro-oncogenic role in Eμ-myc driven B cell lymphoma
Source: Sci Rep. 2016 Nov 25;6:37772. doi: 10.1038/srep37772 (PMC5122906; doi:10.1038/srep37772)
Supplement: Supplementary Material [file srep37772-s1.pdf]

Histone deacetylase 1 plays a predominant pro-oncogenic role in Eμ-*myc* driven B cell lymphoma

Vincent Pillonel[1, 2], Nina Reichert[1], Chun Cao[1], Marinus R. Heideman[1], Teppei Yamaguchi[1], Gabriele Matthias[1], Alexandar Tzankov[3] & Patrick Matthias[1, 2, *]

[1]*Friedrich Miescher Institute for Biomedical Research, Novartis Research Foundation,* 4058 Basel, *Switzerland.* [2]*Faculty of Sciences, University of Basel, 4031 Basel, Switzerland.* [3]*Pathology Institute, University Hospital Basel, 4031 Basel, Switzerland. *Correspondence and requests for materials should be addressed to P.M. (email: patrick.matthias@fmi.ch).*

Supplementary Figure legends

Supplementary Figure 1: B cell specific deletion of *Hdac1* and *Hdac2*. (A) PCR genotyping for *Hdac1* and *Hdac2* wild-type (WT), flox (FL), and knockout (KO) allele and *mb1-cre* transgene (tg). PCR was performed from mouse tails and CD19$^+$ MACS sorted splenic B cells from 8-week-old mice with indicated genotypes. Specific allele annotations are to the left of the gel. Size marker (M) is indicated.

Supplementary Figure 2: Analysis of Eμ-*myc* tg mice lacking Hdac1 and/or Hdac2 in B lymphocytes. (A) Schematic representation of the breeding setup to generate Eμ-*myc* tg mice lacking Hdac1 and/or Hdac2 in B cells. *Hdac1* and *Hdac2* Floxed (F), WT (+) and knockout (Δ) alleles and transgenes (tg) are indicated. Red circle represents visibly enlarged and palpable lymph

nodes scored in the KPLM. **(B)** Schematic representation illustrating the key steps of B cell development in the bone marrow (BM) with the expression of the different cell surface markers listed below. Curved arrows indicate cells in cell cycle. *c-myc* expression in the Eμ-*myc* tg mouse and *mb1* expression in the *Mb1-cre* tg mouse is shown above. **(C)** B lymphocytes from isolated from 8-week-old WT and Eμ-*myc* tg mice were FACS sorted for indicated B cell developmental stages and quantitative real-time PCR (RT-qPCR) was performed for *c-myc*. Data represents means ± s.e.m. (n=3). **(D)** Development of pathologic diseased stage in Eμ-*myc* tg mice. Representative picture of dissected enlarged inguinal lymph nodes (LN), spleen (S), and thymus (T) of Eμ-*myc* tg *Hdac1*$^{+/+}$*;Hdac2*$^{+/+}$, and control WT (*Hdac1*$^{+/+}$*;Hdac2*$^{+/+}$) mice at termination criteria of 1cm LN for KPLM analysis.

Supplementary Figure 3: Predominant overexpression of *HDAC1*, but not *HDAC2*, in BL and DLBCL compared to other cancers. Search for HDAC1 and HDAC2 mRNA expression of human lymphomas with the focus on diffuse large B-cell lymphoma (DLBCL) and Burkitt's lymphomas (BL), two *myc*-driven B cell lymphomas. Online, publically available databases were used. Cancer Cell Line Encyclopedia (CCLE) (www.broadinstitute.org) was used to screen for HDAC1 **(A)** and HDAC2 **(B)** mRNA expression in tumor cell lines. Extracted mRNA expression levels (log2 scale) are shown. Gene expression profiles of *HDAC1* **(C)** and *HDAC2* **(D)**, analyzed on Oncomine microarray database. Analysis of HDAC1 and HDAC2 mRNA levels in lymphoma tissues vs. other cancers (Ramaswamy Multi-cancer statistic datasets).

Supplementary Figure 4: Loss of Hdac1 (Hdac1$^{\Delta/\Delta}$;Hdac2$^{+/+}$) has no impact on PBL at 10 weeks and 20 weeks in Eμ-myc mice. Mice were bled at 10 and 20 week of age and blood was

analyzed in an automated blood analyzer, (Sysmex XT-2000). *Hdac1*$^{\Delta/\Delta}$*;Hdac2*$^{+/+}$ Eμ-*myc* mice had no significant difference in PBL counts at **(A)** 10 and **(B)** 20 weeks compared to control *Hdac1*$^{+/+}$*;Hdac2*$^{+/+}$ Eμ-*myc* mice. Shown are detailed analysis of different Hdac1 knockout animals with or without Eμ-*myc* at 10 and 20, weeks (n≥10). Frequency (%) of PBL of indicated. p-value calculated with the Wilcoxon Signed-Rank Test. Significant differences in means is indicated, $**p < 0.01$; N.S. for not statistically significant.

Supplementary Figure 5: Hdac1 and Hdac2 are not deleted in tumors arising in 4-OHT treated transplanted mice. (**A**) In vivo conditional ablation of Hdac1 and Hdac2 using the CreER-LoxP system inducible with tamoxifen (4-OHT) treatment in Hdac1$^{F/F}$;Hdac2$^{F/F}$; ActinCre tg mice. Recombination was not 100%, since Hdac1 (and Hdac2) floxed alleles did not disappear completely. (**B**) Representative PCR genotyping of tumors from control (Ctr) and tamoxifen (4-OHT) treated transplanted mice. Control Hdac1 and Hdac2 KO PCR from Hdac1$^{\Delta/\Delta}$;Hdac2$^{\Delta/\Delta}$ mice is included. PCR genotyping for Hdac1 and Hdac2 wild type (WT), floxed (FL) and knockout (KO) alleles are indicated to the left of each gel. PCR for Hdac1, Hdac2 and Eμ-myc are to the left of each gels. (**C**) Hdac1 and Hdac2 are expressed in tumors from 4-OHT treated transplanted mice. Immunohistochemistry (IHC) of tumors from Ctr and 4-OHT treated transplanted mice (splenic sections). (**D**) Control IHC experiment for Hdac1, Hdac2 and c-Myc antibodies using Hdac1$^{\Delta/\Delta}$;Hdac2$^{+/+}$ Eμ-myc mice, Hdac1$^{+/+}$;Hdac2$^{\Delta/\Delta}$ Eμ-myc mice and Hdac1$^{+/+}$;Hdac2$^{+/+}$ Eμ-myc mice from serial splenic sections stained for c-Myc, Hdac1, and Hdac2.

Supplementary Figure 6: *Hdac1*$^{\Delta/\Delta}$*;Hdac2*$^{\Delta/+}$ mice have normal numbers of mature B cells in the spleen. (**A**) Spleen cells were obtained from 8-week old mice with indicated genotypes and

stained with B cell surface marker-specific antibodies, including B220, IgM and IgD and analyzed by flow cytometry. Quantification of flow cytometry analysis with s.e.m. (n=3 biological replicates). Average number of absolute B cells ($B220^+$, IgM^+, IgD^+) are shown in bar plots. Statistical analysis was performed with Student unpaired 2-tailed *t* test. N.S., not statistically significant.

Supplementary Figure 7: Full-length immunoblots of Hdac1 and Hdac2 expression. Immunoblots for (**A**) Hdac1, (**B**) Hdac2, and (**C**) actin are shown. (**C**) Hdac1 blot was stripped and reblotted for actin (see residual Hdac1 bands). Mice genotypes are indicated below. Molecular size marker is shown in the first row of each gel. Protein of interest is indicated on the right of each gel.

Supplementary Figures

Suppl. Figure 1

A

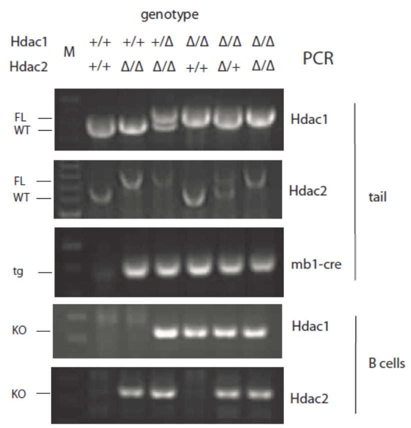

Suppl. Figure 2

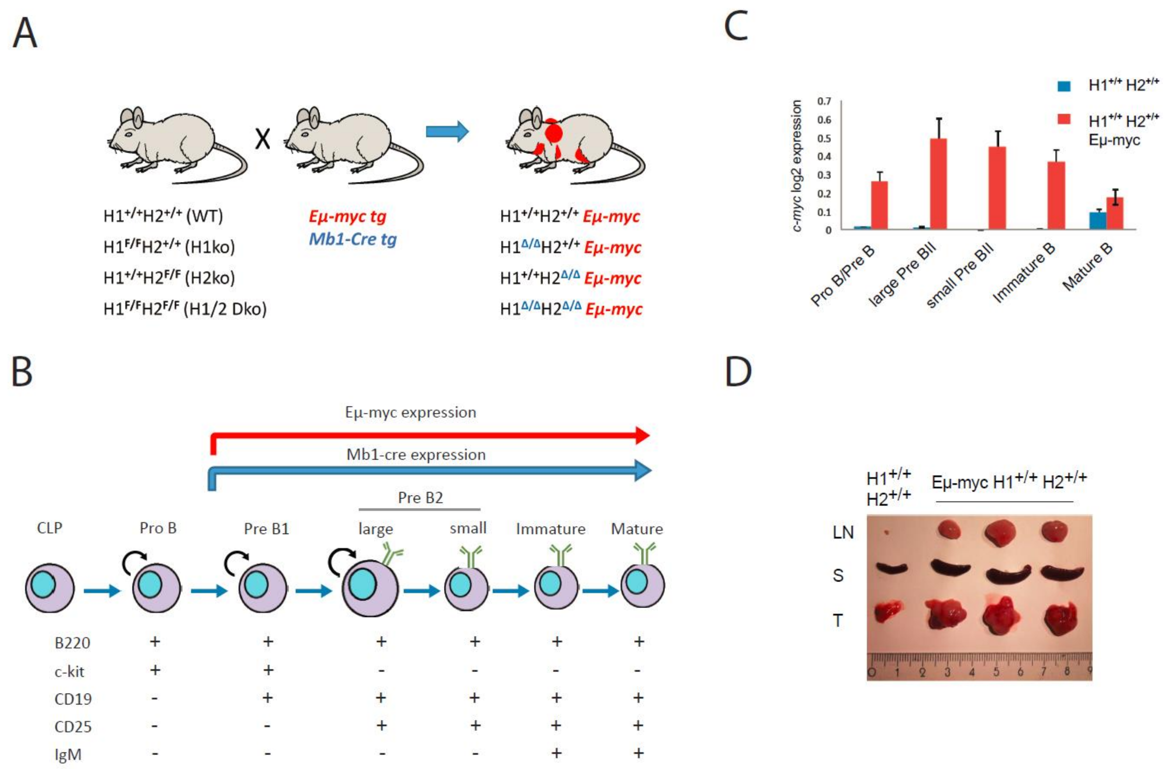

Suppl. Figure 3

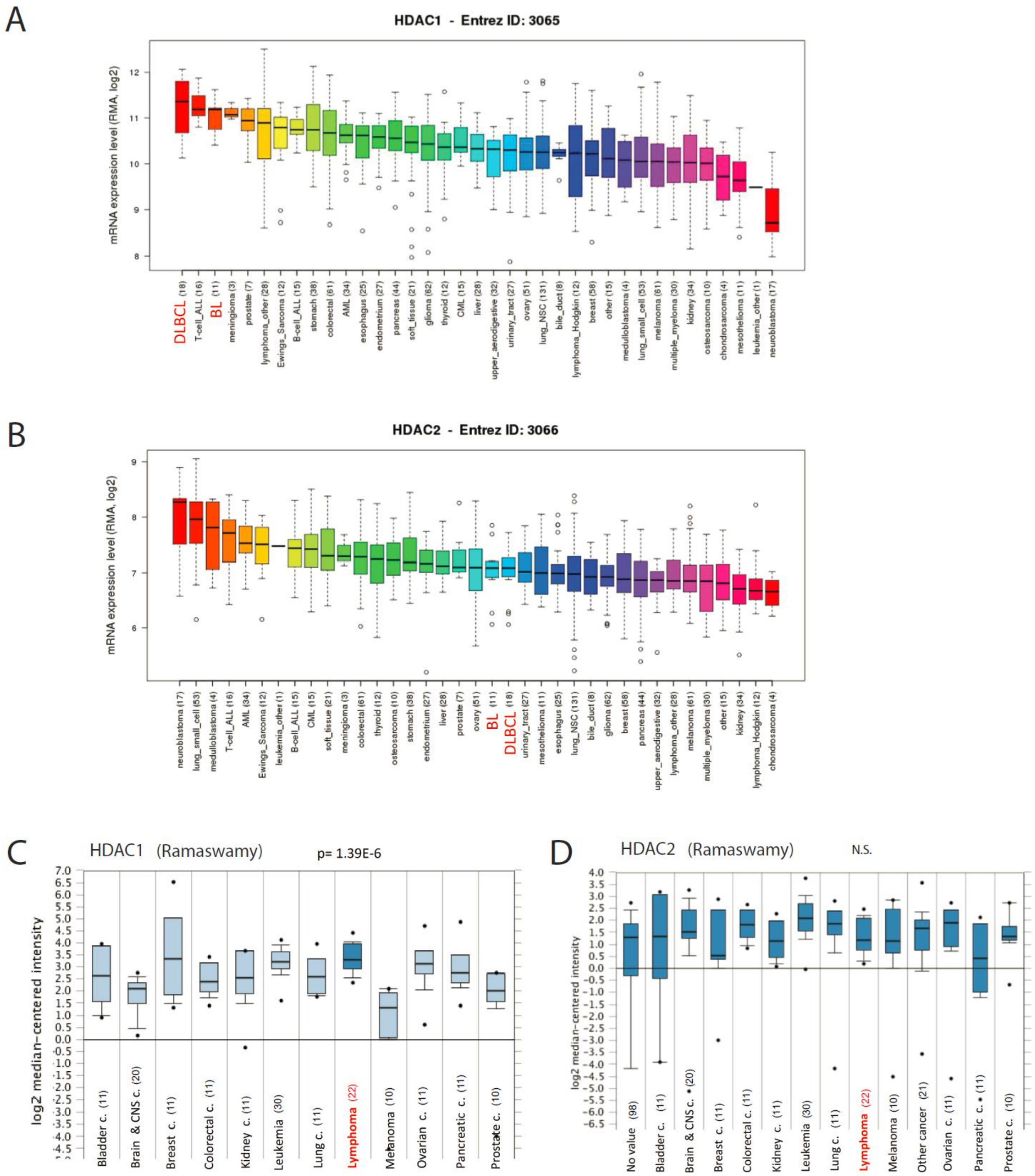

Suppl. Figure 4

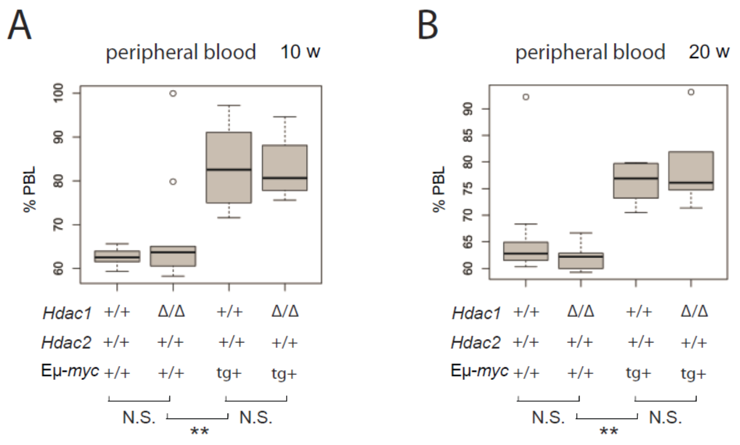

Suppl. Figure 5

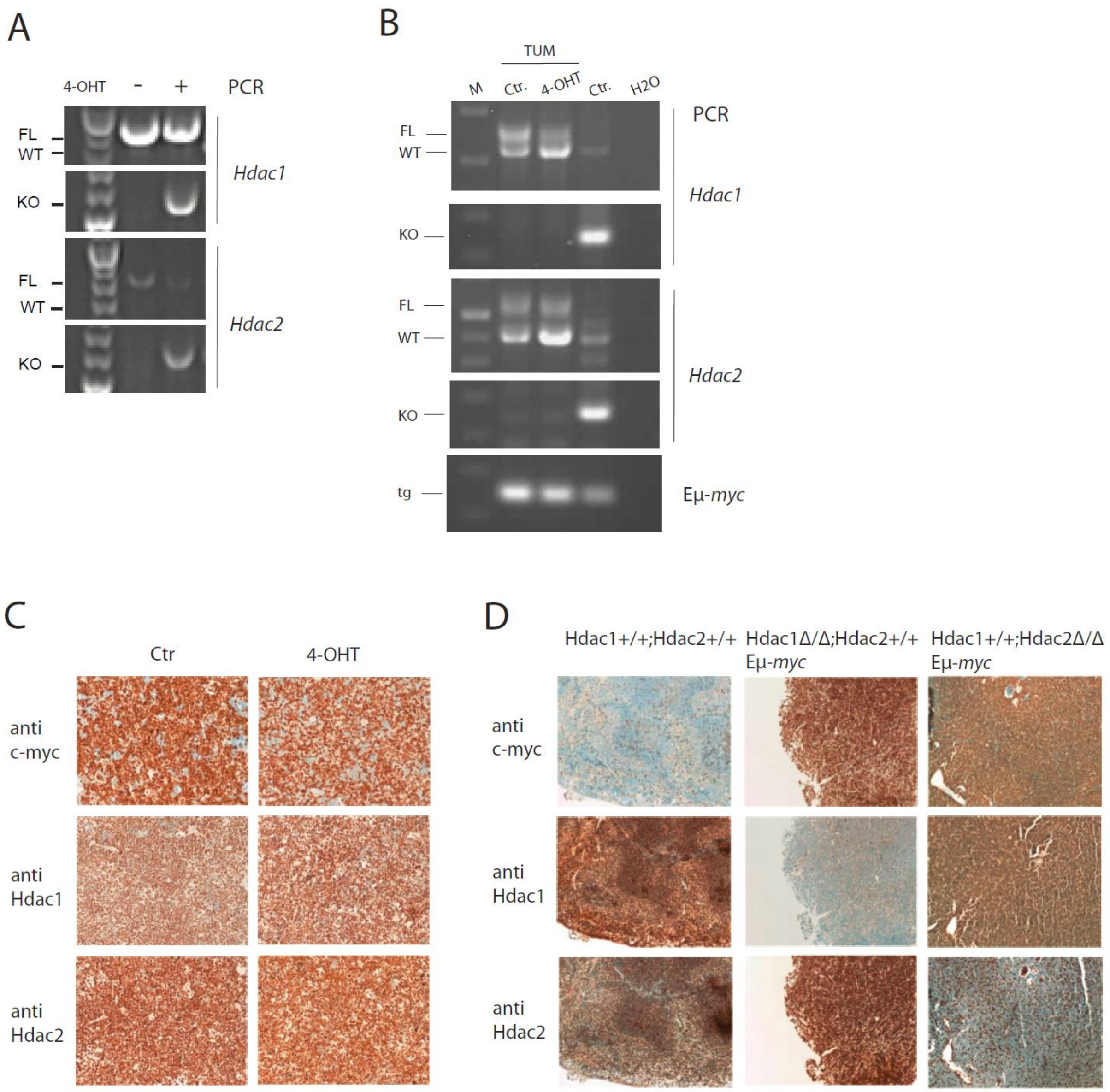

Suppl. Figure 6

A

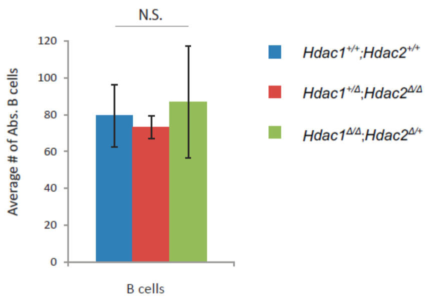

Suppl. Figure 7

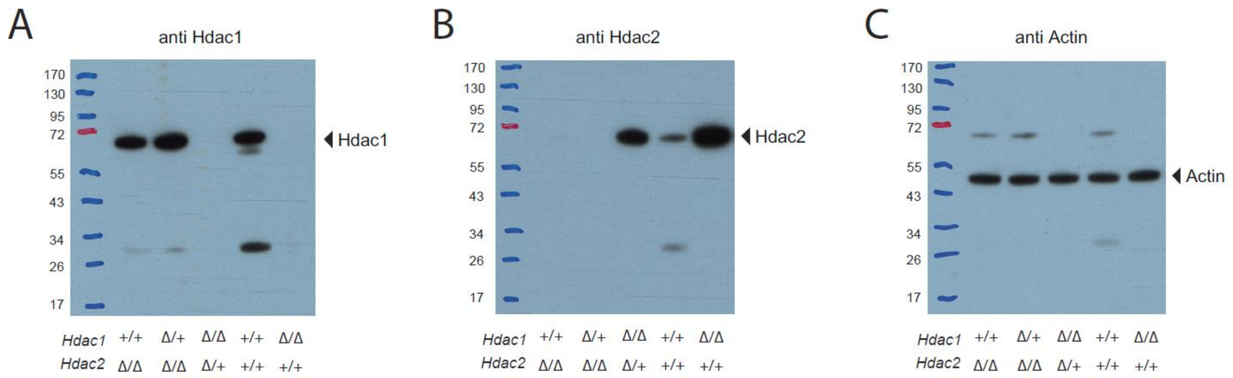

Supplementary methods

Immunohistochemistry (IHC)

Immunohistochemistry experiments were performed on Ventana DiscoveryUltra instrument (Roche Diagnostics, Manheim) with the procedure RUO Discovery Universal. CC1 (Roche Diagnostics, Manheim) pre-treatment was used for anti-HDAC1, -HDAC2, and -c-Myc antibodies with the following incubation times: 16, 24, and 72 min respectively. Antibodies were incubated for one hour at 37°C except for anti-c-Myc which was incubated for 16 min. In addition, a blocking step (Innovex Background Buster NB306, Innovex, 12 min incubation) was added for anti-HDAC1 and -HDAC2 antibodies. Detection of bound anti-HDAC1, -HDAC2 and -c-Myc antibodies was achieved by using anti-rabbit HQ followed by anti-HQ horseradish peroxidase (Roche Diagnostics, Manheim) incubated for 32 min at 37°C. ChromoMap DAB kit (Roche Diagnostics, Manheim) was used for the detection and slides were counterstained with Hematoxylin II and Bluing Reagent (Roche Diagnostics, Manheim) for 8 min. Hdac1 and Hdac2 staining were used to confirm the genotype of the lymphoma.
